# Higher than 60% Dielectric Tunability in Ba_0.6_Sr_0.4_TiO_3_ Films Using TiO_2_ Anatase Buffer Layers

**DOI:** 10.3390/nano15231797

**Published:** 2025-11-28

**Authors:** Pengzhan Zhang, Jiaming He, Xinyu Liu, Leng Zhang, Ling Zhang, Danbei Wang, Kongpin Wu, Sake Wang

**Affiliations:** 1 Jiangsu Engineering Research Center for Digital Intelligent Testing of Integrated Circuits, Jinling Institute of Technology, College of Electronic and Information Engineering, Nanjing 211169, China; 2507050013@stu.jit.edu.cn (J.H.); 2407010008@stu.jit.edu.cn (X.L.); zhangleng2018@jit.edu.cn (L.Z.); zhangling@jit.edu.cn (L.Z.); 00000005182@jit.edu.cn (D.W.); kpwu@jit.edu.cn (K.W.); isaacwang@jit.edu.cn (S.W.); 2 Collaborative Innovation Center of Advanced Microstructures, National Laboratory of Solid-State Microstructures, Nanjing University, Nanjing 210093, China

**Keywords:** Ba_0.6_Sr_0.4_TiO_3_, dielectric tunability, TiO_2_ buffer layer, pulsed laser deposition

## Abstract

In this work, Ba_0.6_Sr_0.4_TiO_3_ (BST) films were deposited on Si(100) and Pt(111)/Ti/SiO_2_/Si(100) substrates using the pulsed laser deposition (PLD) technique. The effects of TiO_2_ buffer layer thickness and preparation temperature on the microstructure and electrical properties of BST films were studied in detail. We intensively investigated the influence of the TiO_2_ buffer layer on the microstructure of BST films by using X-ray diffraction and scanning electron microscopy. We found that anatase crystalline TiO_2_ buffer layers within 15 nm thicknesses could significantly change the BST films from an irregular orientation to the (111) preferential orientation. The TiO_2_ anatase layers could promote the growth of BST film grains for obtaining minimal stress and low lattice distortion, increase the nucleation density, and improve its surface morphology, resulting in higher dielectric constant and resistance voltage, and lower dielectric loss and leakage current density. The dielectric constant, dielectric loss, and dielectric tunability of the BST devices with 8 nm thick TiO_2_ anatase buffer layers at 1 MHz were 856.5, 0.017, and 64.3%, respectively. The achieved high dielectric tunability indicates BST with TiO_2_ anatase buffer layers as one of the encouraging candidates for RF and microwave tunable applications at room temperature.

## 1. Introduction

Tunable microwave devices are key elements in building a new generation of reconfigurable communication systems, which can achieve flexible switching of operating frequencies and rapid scanning of radar beam direction and improve anti-interference capabilities [1,2,3]. However, for devices based on traditional semiconductor materials, their electron migration, driven by the external field, is accompanied by significant Joule heat, making its wide used at higher frequencies difficult. Ferroelectric materials have been developed and have attracted widespread attention from researchers after demonstrating that the dielectric constant changes with a change in the applied electric field [4,5]. As we all know, barium titanate (BaTiO_3_), calcium titanate (CaTiO_3_), strontium titanate (SrTiO_3_) are three typical dielectric materials. Their solid solutions, Barium strontium titanate (Ba*_x_*Sr_1−*x*_TiO_3_, BST), have important application in microelectronic devices, especially dynamic random memory and tunable microwave devices, due to their simple lattice structure, and high dielectric and ferroelectric properties, and have been studied extensively in recent years [6,7,8,9,10,11,12,13,14,15,16,17,18,19,20,21,22,23,24,25,26,27,28,29,30,31,32,33]. Generally, orientation and strain change with the BST film fabrication conditions and parameters (such as doping [6,7], compositing [8,9,10,11,12], electrodes [13,14,15], film thicknesses [16,17,18,19], deposition temperatures [20,21,22,23], buffer layers [24,25,26,27,28], oxygen pressure [22,29], etc.), and the coexistence and competition between these parameters brings challenges to the establishment of the structure–activity relationship and the collaborative optimization of its properties. By studying the relationship between the microstructure of BST films and their properties under different deposition conditions, so as to obtain the intrinsic dielectric properties of the BST films, the quality of BST films can be improved and the dielectric loss of the substrate material can be reduced. Therefore, it is of essential research value and scientific significance to clarify the physical nature of the structure–activity relationship and then obtain BST films with high dielectric tunability and low dielectric loss.

Although the performance of BST has made great progress after more than 20 years of in-depth research, it is still difficult to obtain higher tunability-related suitable dielectric materials that can meet the requirements of high-performance device design, and an important reason is that complex factors such as defects, domain structure or wall, phase structure, or boundaries in the film will have a significant impact on its dielectric properties. When growing a film on a Si substrate, a SiO_2_ insulating layer is first formed on its surface, and Ti is used as an attachment layer for the Pt bottom electrode, so it is more important to grow (111)-oriented (Ba,Sr)TiO_3_ films on Pt/Ti/SiO_2_/Si substrates. However, BST films deposited directly on Pt/Ti/SiO_2_/Si substrates are generally polycrystalline structures with relatively low dielectric constants and tunability. Fardin et al. and Liu et al. reported that the dielectric tunability of Ba_0.6_Sr_0.4_TiO_3_ films deposited on (111)-oriented sapphire single crystal and (00*l*)-oriented (LaAlO_3_)_0.3_(Sr_2_TaAlO_6_)_0.7_ single crystal substrates reached 64% and 57%at at high frequencies, respectively. They attribute the improvement in dielectric tunability to the good lattice matching between the single crystal substrate and BST films, which is easy to grow with preferential orientation and have improved dielectric properties [19,23]. However, the monocrystalline substrate is more expensive, and the requirement of compatibility with integrated Si circuits makes it more practical to prepare high-quality BST films on Si substrates. In order to obtain higher-quality films on Pt/Ti/SiO_2_/Si substrates, buffer layers (such as MgO [24,25], Bi [26], La_0.7_Sr_0.3_CoO_3_ [27], TiN [28], etc.) have been selected to improve its microstructure and dielectric properties. Zhu et al. and Kim et al. improved the dielectric properties of BST films deposited on Si substrates by adding 5 nm thick MgO and 50 nm thick TiO_2_ buffer layers and obtained 30% and 33.2%improved dielectric tunability, respectively [25,33]. However, the obtained dielectric tunability in this case is relatively low compared to the BST grown on single crystal substrates and are still far from use in applications.

TiO_2_ films have been used as buffer layers to prepare (Ba,Sr)TiO_3_ [32,33], Pb(Zr,Ti)O_3_ [34,35,36], (Pb,Sr)TiO_3_ [37], CaBi_4_Ti_4_O_15_ [38], Bi_4_Ti_3_O_12_ [39], etc. Kim et al. pointed out that 50 nm thick TiO_2_ anatase buffer layers grown by the ALD method on high resistivity Si substrates followed by the Pulsed Laser Deposition of Ba_0.6_Sr_0.4_TiO_3_ thin films onto a TiO_2_ buffer layer can improve the crystallinity and dielectric properties of BST films [32,33]. Muralt et al. used the sputter deposition method to use a TiO_2_ film with several nanometers thickness as a buffer layer and pointed out that the rutile phase crystalline TiO_2_ film can make the PZT film oriented (111), while amorphous TiO_2_ has no effect on the film orientation [34,36]. Chen et al. reported that the TiO_2_ buffer layer crystallized as the rutile (110) and anatase (101) with a thickness of nearly 5 nm grown by the Sol–Gel method was critical for improving the crystallinity and surface morphology of both the thinner (40 nm) and thicker (330 nm) PST films, which exhibited a (l00) preferred orientation [37]. However, they did not research the influence of TiO_2_ buffer layer thickness and crystallization on the microstructure and dielectric properties in detail, and the related exciting results of improved dielectric tunability are still lacking in the open literature.

In this paper, the effects of TiO_2_ buffer layer thickness and preparation temperature on the microstructure and electrical properties of Ba_0.6_Sr_0.4_TiO_3_ (BST) films were studied in detail. The BST films were successfully deposited on the Si(100) and Pt/Ti/SiO_2_/Si(100) substrates using TiO_2_ as the buffer layer. Through XRD experiments, we found that the anatase phase crystalline TiO_2_ buffer layer changes the BST film lattice structure from an irregular orientation to the (111) preferential orientation. Then, we analyzed the effect of a TiO_2_ anatase buffer layer on improvements in the electric properties of BST films. The dielectric constant of BST was relatively increased, while the dielectric loss was greatly reduced after the addition of the ultra-thin TiO_2_ anatase buffer layers. The I-V characteristics of the corresponding samples show that the surface roughness of the BST films improved well with the addition of the TiO_2_ anatase buffer layer, so the leakage current was reduced accordingly. Compared with previous reports of TiO_2_-buffered BST research, we confirmed that an ultra-thin TiO_2_ anatase buffer layer could promote the growth of BST film grains for obtaining minimal stress and low lattice distortion, increasing the nucleation density and improving its surface morphology, resulting in higher dielectric constants and resistance voltage, and lower dielectric loss and leakage current density. The results obtained in this work are the groundbreaking reports of the improved dielectric properties from the ultra-thin TiO_2_-buffered (111) orientated BST films deposited by using pulsed laser deposition (PLD). The dielectric constant, dielectric loss, and dielectric tunability of BST films with an 8 nm thick TiO_2_ anatase buffer layer at 1 MHz were 856.5, 0.017, and 64.3%, respectively. The obtained high tunable dielectric characteristics of BST devices have important scientific significance and application value in realizing high-speed, low-power, wide-band RF, and microwave tuning circuits.

## 2. Materials and Methods

### 2.1. Material Fabrication

The BST and TiO_2_ films were deposited on Si(100) and Pt/Ti/SiO_2_/Si(100) substrates by using the PLD technique with a KrF ns-pulsed laser (λ_exc_ = 248 nm, pulse width ∼20 ns, repetition rate = 5 Hz, and energy density = 2 J/cm^2^), respectively. BST targets were prepared by the conventional solid-state reaction technique, and high-purity metal Ti targets were used to prepare TiO_2_ films. The substrates were seriatim-cleaned in a (NH_4_OH+H_2_O_2_) solution and a buffered (HF+H_2_O_2_) solution, respectively. After the substrate was placed into the reaction chamber, the vacuum was pre-pumped to 5 × 10^−4^ Pa using a turbomolecular pump, and the substrate was heated to a specific temperature and remained stable during the whole deposition processes. Before deposition, the substrates were pretreated with argon gas glowing for 1 min at room temperature, with an RF power of 30 W under a reaction pressure of 10 Pa. After the pretreated process, high-purity oxygen was then introduced and the working pressure during precipitation was maintained with an oxygen flow rate of 10.0 sccm. We first prepared a TiO_2_ buffer layer with a thickness ranging from 4 nm to 40 nm under 10 Pa oxygen pressure and different deposition temperatures (200–650 °C). Next, BST films (360 nm) were deposited in situ on the TiO_2_ buffer layer under the condition of 15 Pa oxygen pressure and substrate temperature of 650 °C in the same vacuum chamber with a deposition rate of 1.8 Å/s. After deposition, the films were cooled in situ and slowly dropped to room temperature at a rate of 5 °C/min in an oxygen environment of 1 atm. For comparison, BST films without buffer layers were also prepared under the same preparation conditions.

### 2.2. Characterization

The films were structurally characterized and analyzed by an X-ray diffraction (XRD, Bruker D8 Endeavor, Berlin, Germany), a scanning electron microscopy (SEM, Hitachi S-4800, Tokyo, Japan), and a scanning probe microscope (SPM, NT-MDT Solver P47-PRO, Moscow, Russia) operating in the contact atomic force microscope (AFM) mode, respectively. The grain size along the (111) orientation and the lattice parameter of BST films with various TiO_2_ thicknesses were calculated using Scherrer’s formula and Bragg’s Law [14] from XRD spectra, both of which are listed in Table 1. The thicknesses of the BST films were measured with an ET350 Talysurf profilometer (Kosaka Laboratory Ltd., Japan) and verified with cross-sectional SEM, which were all precisely controlled by adjusting the deposition time. In order to measure the electrical properties, Pt/Ti/SiO_2_/Si were used as the bottom electrodes of BST films, and dot Pt electrodes with a diameter of about 0.13 mm were plated on the surface of BST samples by the magnetron sputtering method to form a MIM capacitor structure. Before depositing the Pt electrode, a stainless-steel round hole mask (pore size of 0.13 mm) was placed on the upper surface of BST films. When the mask was placed, part of the films was exposed to the outside of the mask, which can be used as the bottom electrode. The dielectric properties of a BST MIM capacitor were tested and characterized by a HP4284 LCR meter (Agilent, USA) in the frequency range from 1Hz to 1MHz, mainly measuring the dielectric constant and dielectric loss relationship with frequency, and capacitance–voltage (C-V) characteristics. The I-V characteristics were measured by using a Keithley 6517A electrometer (Tektronix, USA) as a voltage source and a picoampere meter.

## 3. Results and Discussion

### 3.1. X-Ray Diffraction Spectrum (XRD)

**Figure 1:**
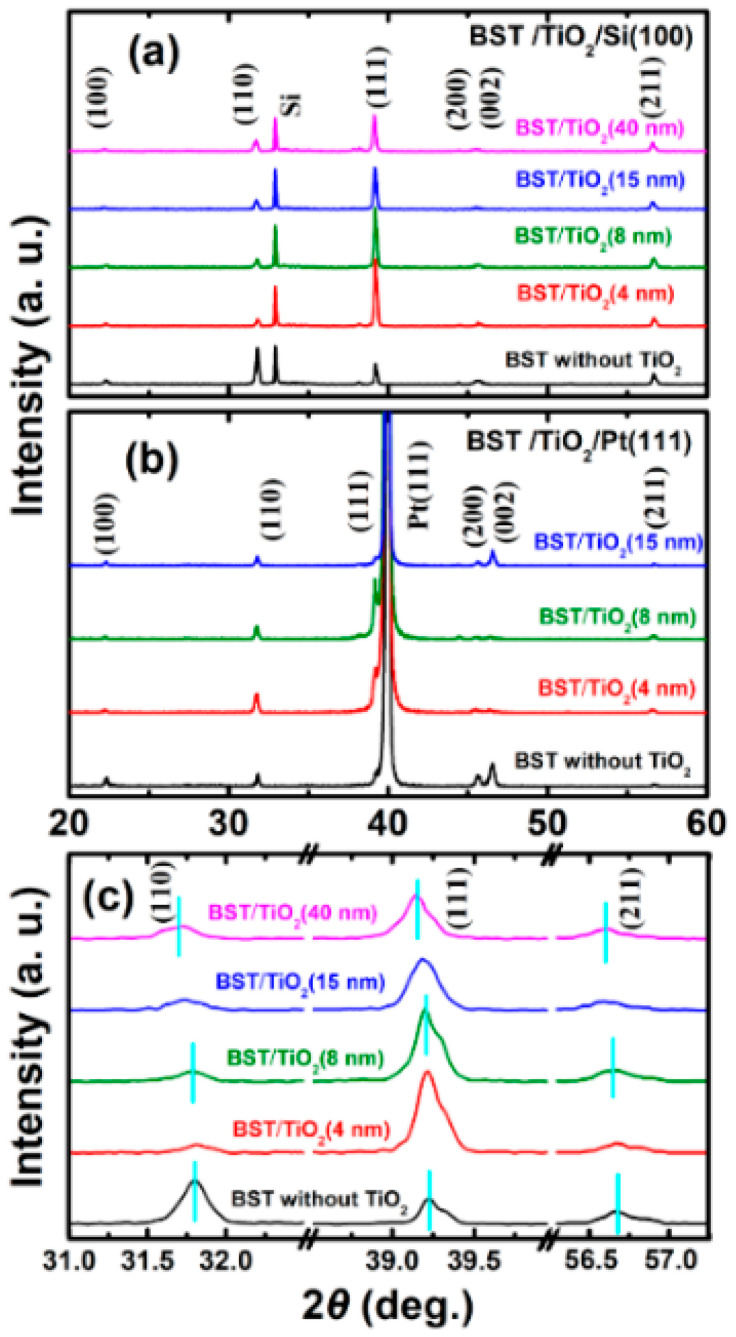
(**a**,**b**) XRD patterns of BST-buffered TiO_2_ with different thicknesses deposited on Si(100) and Pt(111)/Ti/SiO_2_/Si(100) substrates, respectively. (**c**) Enlarged XRD patterns of BST film-buffered TiO_2_ with different thicknesses deposited on Si(100) substrates.

Figure 1 shows the XRD patterns of BST films prepared on Si(100) and Pt(111)/Ti/SiO_2_/Si(100) substrates, without and with TiO_2_ buffer layers of different thicknesses, respectively. All diffraction peaks are indexed according to the standard XRD data of BaTiO_3_ powder. The TiO_2_ films were fabricated at a substrate temperature of 650 °C, with thicknesses of 4 nm, 8 nm, 15 nm, and 40 nm, respectively. As can be seen from Figure 1, the BST films without buffer layers exhibit typical perovskite polycrystalline structures with an irregular orientation. Due to the fact that the TiO_2_ buffer layer serves as the initial nucleation and growth template for BST films and interacts and reacts with the BST films during their growth process, no TiO_2_ crystallization peaks are observed, which is consistent with the relevant literature [35,37,38,39]. From Figure 1a, it can be observed that after incorporating a TiO_2_ buffer layer with a relatively small thickness (0–15 nm), the BST films prepared on the Si(100) substrate show a (111) preferential orientation. However, the (111) peak begins to weaken when the thickness reaches 15 nm, and the preferential orientation of the BST film becomes less pronounced when the thickness of the buffer layer reaches 40 nm. For the BST films prepared on the Pt(111)/Ti/SiO_2_/Si(100) substrates (Figure 1b), significant changes also occur in the XRD patterns after the incorporation of a TiO_2_ buffer layer between the substrates and BST films.

From the XRD patterns, we can observe that the poly-crystalline BST thin films are dominated by three principal orientations: (110), (111), and (211). Figure 1c presents an enlarged XRD pattern of the corresponding samples in Figure 1a on the Si(100) substrates in the 2*θ* range of 31–57.5°. The correspondence peak positions and F.W.H.M. of (110), (111), and (211) are listed in Table 2. Here, we use the Lotgering method to estimate the extent of the three principal orientations by XRD integrated intensity [40].

Regarding the Lotgering orientation factor (F) of the oriented BST films, we define it as follows:

(1) $$F\;=\;\frac{P-P_{0}}{1-P_{0}}$$

The Lotgering orientation factor (F) (Equation (1)) is given by Equations (2) and (3) as follows:

(2) $$P\;=\;\frac{I_{OBS}}{\Sigma I{\left(hkl\right)}_{OBS}}$$

(3) $$P_{0}=\frac{I_{ISO}}{\Sigma I{\left(hkl\right)}_{ISO}}$$

where *P* is defined as the proportion of the relative integrated intensities of the actual measured oriented peak reflection ((110), (111), or (211)), *I*_OBS_, to the sum of the relative integrated intensities of all (*hkl*) reflections in the TiO_2_-buffered BST films (Σ*I*(*hkl*)_OBS_). *P*_0_ is defined as the proportion of the related isotropic integrated intensity of special plane ((110), (111), or (211)), *I*_ISO_, to the summation of the relative integrated intensities of all (*hkl*) reflections when fully oriented for non-textured BST films without TiO_2_ (Σ*I*(*hkl*)_ISO_).

**Figure 2:**
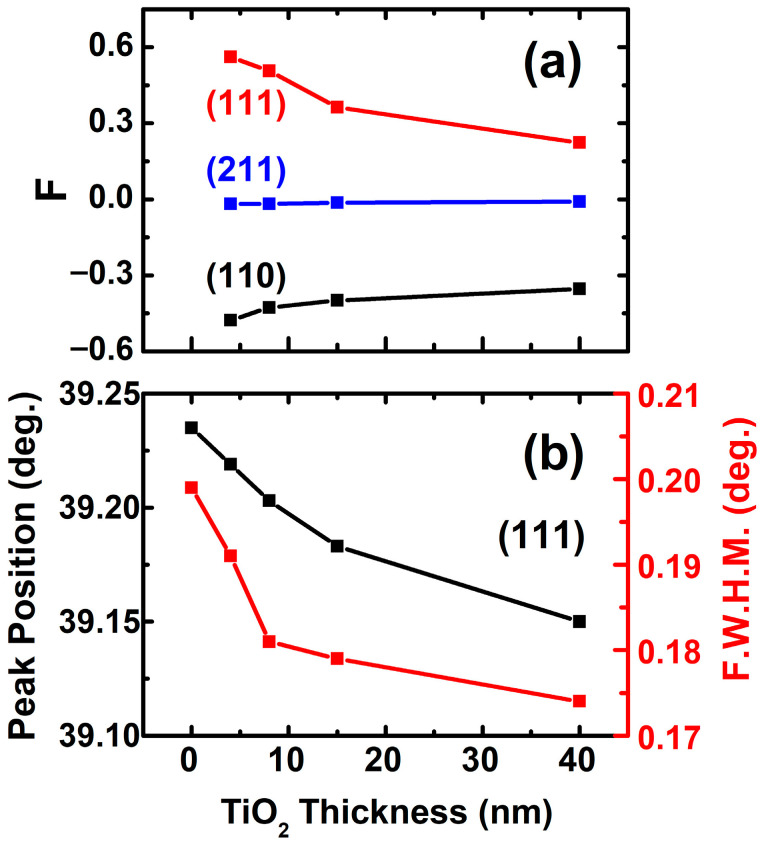
(**a**) The Lotgering orientation factor (F) of three principal orientations: (110), (111), and (211); (**b**) peak positions and *F.W.H.M.* of the (111) preferential orientation in BST films with TiO_2_ buffer layers of different thicknesses.

Figure 2a shows the Lotgering orientation factor changed with TiO_2_ buffer layers of different thicknesses. As the TiO_2_ thickness increases from 4 nm to 40 nm, the Lotgering orientation factor of (111) decreases from 0.56 to 0.22, while the Lotgering orientation factor of (110) and (211) are negative and increased. These results suggest that all the BST films with TiO_2_ buffer layers ranged from 4 nm to 40 nm have highly (111) preferential orientations.

When the TiO_2_ buffer layer is very thin (4–8 nm), the intensity of the (111) peak is significantly stronger compared to that of the pure BST films, and the BST films exhibit a (111) preferred orientation. However, when the thickness of the buffer layer increases to 15 nm, the intensity of the (111) peak weakens. These results are similar to the case of direct deposition on the Pt(111)/Ti/SiO_2_/Si(100) substrate in Figure 1b. Since the ultra-thin TiO_2_ buffer layer serves as the initial nucleation and growth template for BST films and interacts with the BST films during their growth process, the crystallization structure of the BST films changes, resulting in a (111) preferential orientation. This is consistent with relevant literature reports on other films prepared with TiO_2_ as a buffer layer [34,35,36,38]. Therefore, it can be concluded that ultra-thin TiO_2_ buffer layers have an impact on the crystallization orientation of BST films.

From Figure 1c and Table 2, it is worth noting that all the XRD peaks of BST films shifted slightly to lower-angle sides after incorporating a TiO_2_ buffer layer and thus the expansion of a lattice. Furthermore, it can also be observed that the F.W.H.M. of the most intense diffraction peak along the (111) orientation narrow down after incorporating TiO_2_ buffer layers, indicating better crystallinity as compared to that of BST without TiO_2_, as shown in Figure 2b and Table 2.

### 3.2. The Effect of the Lattice Structure of a TiO_2_ Buffer Layer on the Structure of BST Films

**Figure 3:**
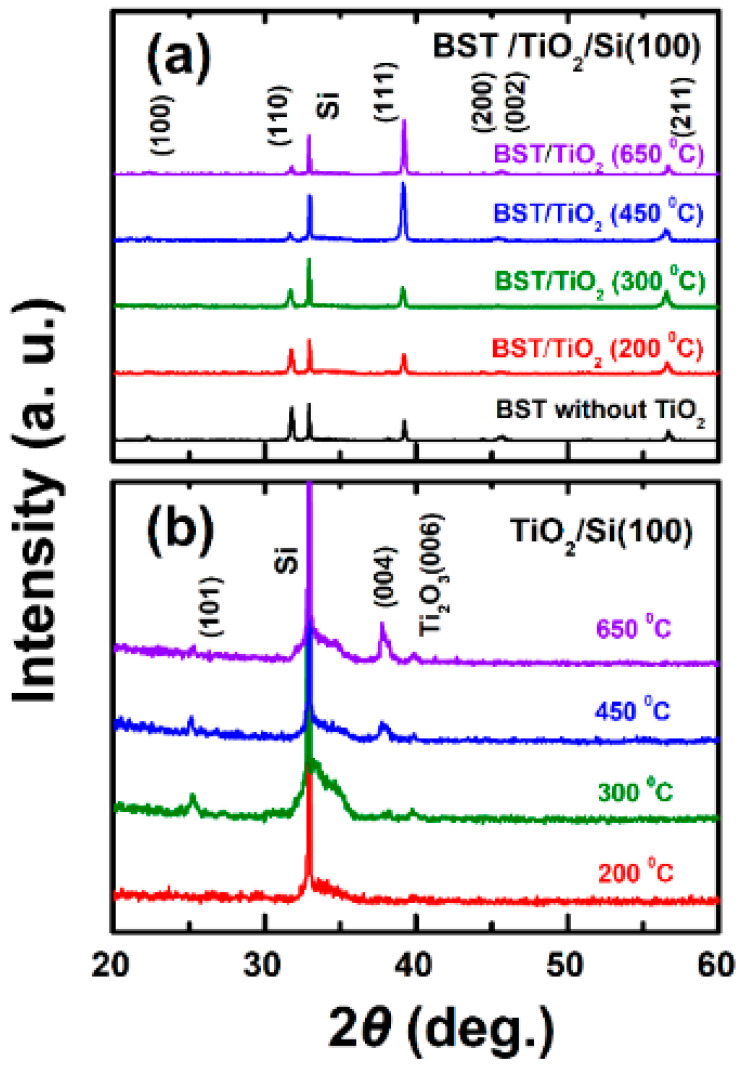
XRD patterns of (**a**) BST thin films deposited on TiO_2_ buffer layers which were fabricated under various temperatures; (**b**) TiO_2_ thin films deposited under various temperatures.

To understand the effect of lattice structure of a TiO_2_ buffer layer on the structure of BST films in depth, we intensively investigate the crystal structure and composition of a TiO_2_ buffer layer by performing XRD tests. Figure 3a shows the XRD patterns of BST films deposited on TiO_2_ buffer layers (8 nm) prepared at different temperatures. All the BST films were fabricated at a substrate temperature of 650 °C. We can observe that the TiO_2_ buffer layers prepared at 200 °C and 300 °C have almost no impact on the growth of the BST films. However, at 450 °C and 650 °C, the intensity of the (111) peak of the BST films significantly increases, while the intensities of other diffraction peaks are relatively weaker. This indicates that the TiO_2_ buffer layer prepared at higher temperatures can transform the BST films from an irregular orientation to the (111) preferential orientation, whereas the TiO_2_ buffer layer prepared at lower temperatures has almost no effect on the lattice structure of BST films.

Then, we investigate the crystal structure and composition of the TiO_2_ buffer layers by performing XRD under high-power measurement conditions (200 mA, 50 kV). As shown in Figure 3b, no obvious peaks are observed when the substrate temperature is 200 °C, indicating that the TiO_2_ film is amorphous. At a substrate temperature of 300 °C, a very weak (101) peak of the anatase phase of TiO_2_ appears, suggesting insufficient crystallization of the film. When the temperature is increased to 450 °C, both the (101) and (004) peaks of the anatase phase are present [41,42,43,44], with a significant increase in peak intensity compared to that at 300 °C, indicating that TiO_2_ has crystallized. As the temperature continues to rise to 650 °C, the crystallization peaks of the anatase phase become stronger compared to those at 450 °C. It is noteworthy that no rutile phase of TiO_2_ is detected. Thus, it can be concluded that the TiO_2_ buffer layer grown below 300 °C is basically amorphous, while that grown above 450 °C is crystalline.

Combined with Figure 3a, it can be inferred that the crystalline anatase-phase TiO_2_ buffer layer enables the (111) preferential orientation of BST films. After incorporating the crystalline anatase-phase TiO_2_ buffer layer, we can determine that the grain size along the (111) orientation and lattice parameter of BST films increases with an increase in the thickness of the TiO_2_ buffer layer because of the shift in the XRD peak to lower-angle sides and thus the expansion of the lattice, as shown in Figure 1c and Figure 2b, and Table 1 and Table 2. Compared with the lattice parameter of the BST films without a buffer layer (a = b = c = 3.9670 Å), the lattice parameter of the TiO_2_-buffered BST films increased to 3.9681 Å, 3.9695 Å, 3.9744 Å, and 3.9792 Å with TiO_2_ thicknesses of 4 nm, 8 nm, 15 nm, and 40 nm, respectively.

Generally, the differences in lattice parameters of various lattices would lead to tensile or compressive stresses in the film deposition as a result of lattice misfit. To estimate the lattice misfit in our BST films, we calculated the lattice strain along the (111) preferential orientation of the tensile stress, which is listed in Table 2. As the thickness of the TiO_2_ buffer layer increases, the lattice strain of the films decreases from 0.0026 to 0.0021. It can be concluded that the crystalline anatase-phase TiO_2_ buffer layer plays an important role in the deposition of texture BST thin film for obtaining minimal stress and low lattice distortion. This finding is similar to the previous work reported by Muralt regarding the effect of the TiO_2_ buffer layer on the crystal orientation of PZT films [34,36]. Therefore, ultra-thin anatase phase TiO_2_ buffer layers can adhere well to the Pt surface, minimizing the surface energy required for the growth of BST nuclei along the (111) direction, ultimately resulting in a (111) preferred orientation of the films.

### 3.3. Surface Morphology and RMS Surface Roughness

**Figure 4:**
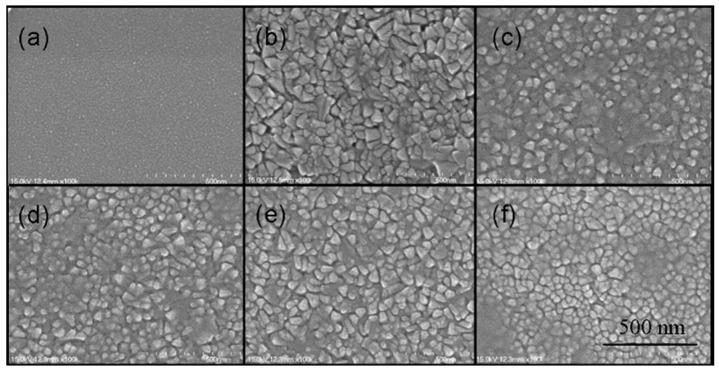
SEM micrographs of TiO_2_ buffer layer and BST films deposited on Pt(111)/Ti/SiO_2_/Si(100) substrates, (**a**) TiO_2_ films; (**b**) BST films without TiO_2_; (**c**–**f**) BST with 4 nm, 8 nm, 15 nm, and 40 nm TiO_2_ buffer layers, respectively.

To further investigate the effect of the TiO_2_ buffer layer on the microstructure and crystallization of the BST films, we performed the SEM patterns of the BST films without and with a TiO_2_ buffer layer in Figure 4. Figure 4a shows the surface morphology image of an 8 nm thick TiO_2_ film prepared at 650 °C. As can be seen from the image, the surface of the TiO_2_ film is flat and dense. Figure 4b shows the SEM image of a BST film without a buffer layer. In terms of grain growth and surface morphology, the BST films directly grown on a Pt substrate exhibit a low nucleation density and a rough surface. The grain sizes of the film vary, with dimensions approximately ranging from 50 to 100 nm. Figure 4c–f show the SEM images of BST films after incorporating TiO_2_ buffer layers of different thicknesses (4 nm, 8 nm, 15 nm, and 40 nm), respectively. It can be observed that after adding TiO_2_ buffer layers between the substrate and the BST films, the grains of the BST film become relatively more uniform, with sizes approximately around 50 nm. Aoki et al. reported that Ti plays a crucial role in the grain nucleation process of perovskite films, and a Ti layer with a thickness of a few nanometers on a Pt substrate can significantly enhance the grain nucleation density [45]. Here, we can consider this Ti layer as a layer of TiO_2_ on the Pt surface. Therefore, depositing a TiO_2_ buffer layer on a Pt substrate can promote the grain growth of BST films, thereby increasing their nucleation density and improving their surface morphology, which is similar to reports on other films prepared with TiO_2_ as a buffer layer [34,36,39]. This results in a denser and smoother surface, forming a BST film with fine and uniform particles.

**Figure 5:**
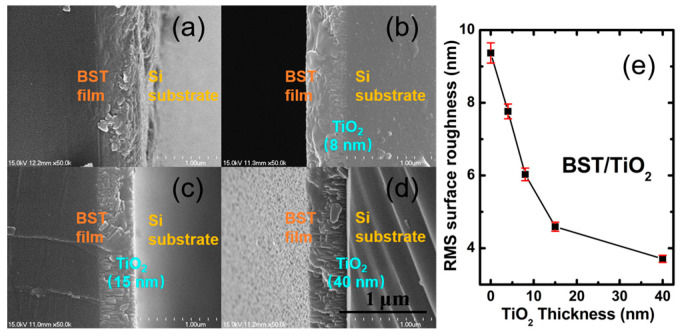
Cross-section SEM of BST films deposited on Si(100) substrates, (**a**) BST films without TiO_2_; (**b**–**d**) BST with 8 nm, 15 nm, and 40 nm TiO_2_ anatase buffer layers, respectively. (**e**) RMS surface roughness with a relative uncertainty of BST films vs. TiO_2_ thicknesses.

To identify the grain shape, interface, and surface roughness of BST-buffered TiO_2_ with different thicknesses, we obtained cross-section SEM and AFM spectra, respectively. Figure 5a–d present the cross-section SEM of BST without TiO_2_ and with 8 nm, 15 nm, and 40 nm TiO_2_ anatase buffer layers, revealing an average thickness of 360 nm for the BST films. The BST films with no buffer layer consist of well-developed grains, and the shape is irregular and plate-like, while the BST-buffered TiO_2_ tends to show columnar growth and the grain shape changes to regular and granular-like. It is worth noting that, after incorporating an ultra-thin (4–8 nm) TiO_2_ buffer layer, the TiO_2_ seems to disappear as the BST films form, indicating that the TiO_2_ anatase buffer layer does not act as a barrier layer but rather serves as the initial nucleation layer for BST, which is similar to that in previous work [39]. The BST and TiO_2_ films adhere well to the substrates with a clear interface between films and substrates, and the BST films were uniform. From the AFM spectra, the RMS surface roughness of BST decreases from 9.37 nm to 3.71 nm when the thickness of the TiO_2_ anatase buffer layer increases to 40 nm, as shown in Figure 5e.

### 3.4. The Effect of a TiO_2_ Buffer Layer on the Dielectric Characteristics

**Figure 6:**
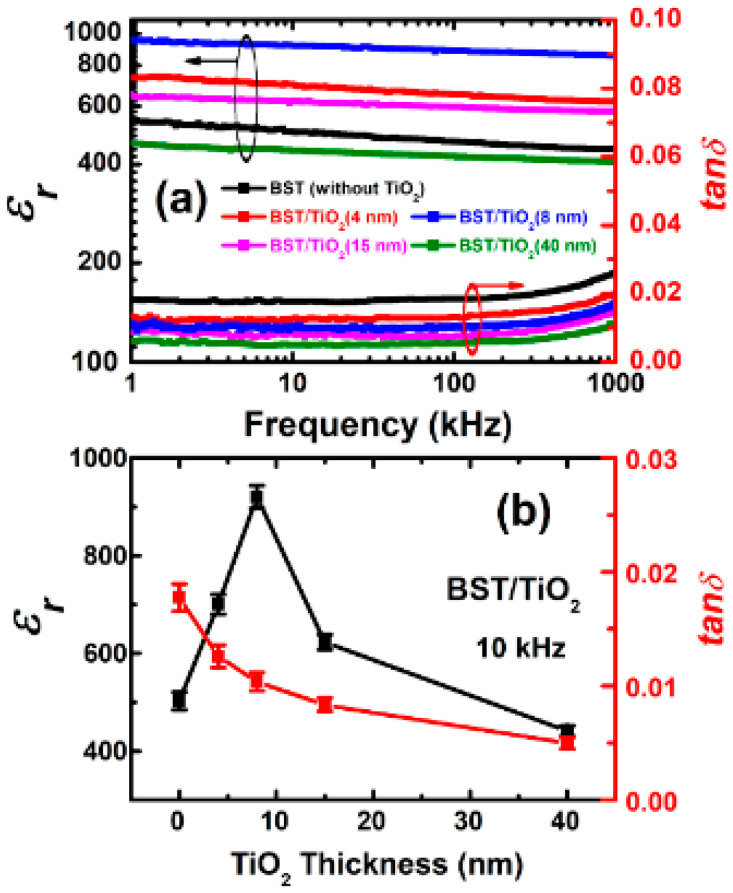
(**a**) Dielectric constant (ε_r_) and dielectric loss (tanδ) as a function of the measuring frequency of BST films without and with 4 nm, 8 nm, 15 nm, and 40 nm TiO_2_ buffer layers, respectively. (**b**) ε_r_ and tanδ with a relative uncertainty of BST films vs. TiO_2_ thicknesses at 10 kHz.

Figure 6a presents the curves of the dielectric constant (ε_r_) and dielectric loss (tanδ) of corresponding BST films prepared on the Pt(111)/Ti/SiO_2_/Si(100) substrates as a function of frequency at room temperature. All the ε_r_ and tanδ values with a relative uncertainty of corresponding BST films measured at 10 kHz are shown in Figure 6b and listed in Table 1. From Figure 6, it can be observed that without a TiO_2_ buffer layer, the ε_r_ and tanδ of the Pt/BST/Pt capacitor are 502.6 and 0.018 at 10 kHz, respectively. Meanwhile, the dielectric constant gradually decreases with an increase in frequency (showing significant frequency dispersion). This result is similar to that of previous works [10,18,21,22]. After incorporating an ultra-thin (4–8 nm) TiO_2_ anatase buffer layer, the dielectric constant of the BST films significantly increases over the entire frequency measurement range, while the loss decreases. As shown in Figure 6b, for the BST with an 8 nm thick TiO_2_ buffer layer, the ε_r_ and tanδ at 10 kHz are 920.3 and 0.010, respectively. When a 15 nm thick buffer layer is added, the dielectric constant decreases compared to that with an 8 nm thick crystalline TiO_2_ buffer layer but remains slightly higher than that of BST films without TiO_2_. However, it is noteworthy that the frequency dispersion becomes relatively smaller at this time. After adding a 40 nm thick TiO_2_ buffer layer, the dielectric constant further decreases and is lower than that of BST films without TiO_2_. Nevertheless, its frequency dispersion and loss are much smaller compared to those of BST films without TiO_2_, with a tanδ of 0.004 at 10 kHz. The above phenomena indicate that the TiO_2_ buffer layer can significantly improve the dielectric properties of the BST films. Simultaneously, the TiO_2_ buffer layer promotes the grain growth of the BST film, greatly increasing the grain nucleation density and resulting in a film with fine particles [32,34,39], thereby reducing its dielectric loss. When the TiO_2_ buffer layer is relatively thick (40 nm), the total dielectric constant decreases instead. This is because TiO_2_ has a relatively low dielectric constant [41]. For a BST film with thick TiO_2_ buffer layers, it cannot be regarded as a simple superposition of the two but should be considered as a series capacitor composed of a Pt/BST/Pt capacitor and a Pt/TiO_2_/Pt capacitor [25].

### 3.5. The Capacitance–Voltage (C-V) Characteristics and Dielectric Tunabilities

**Figure 7:**
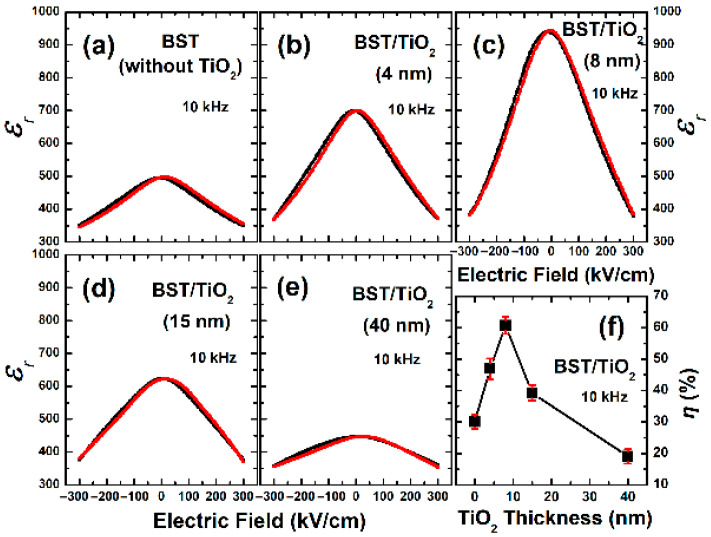
(**a**–**e**) Dielectric constant (ε_r_)—electric field curves at the frequency of 10 kHz of BST films without TiO_2_ and with 4 nm, 8 nm, 15 nm, and 40 nm TiO_2_ buffer layers, respectively. (**f**) The responsible dielectric tunability (*η*) with a relative uncertainty of BST films vs. TiO_2_ thicknesses at 10 kHz under 300 kV/cm. The black and red lines in (**a**–**e**) represent that the bias voltage was scanned through the cycle from a negative value to a positive value and the cycle from a positive value back to a negative value, respectively.

Figure 7a–e show the curves of dielectric constant variation with bias voltage for the corresponding BST films at room temperature. During the measurement processes, the bias voltage was scanned through a cycle from a negative value to a positive value and then back to a negative value, ranging from −300 kV/cm to 300 kV/cm, with a test frequency of 10 kHz. The horizontal coordinates at the centers of the curves are basically located at 0 kV/cm. It is worth noting that BST capacitors showed the absence of splitting and no significant double peaks are observed in any of the C-V curve by testing fifteen related samples, characterizing the paraelectric nature but not the typical butterfly shape from ferroelectric properties.

Regarding the dielectric tunability (*η*) of BST films, we define it as follows [1]:

(4) $$\eta \;(\%)\;=\;\frac{\left(\varepsilon_{0}-\varepsilon_{v}\right)}{\varepsilon_{0}}\times 100\%$$

where *ε*_0_ and *ε_v_* represent the magnitudes of the dielectric constant when the applied electric field is 0 kV/cm and 300 kV/cm, respectively. Based on the dielectric tunability formula (4), we calculated that the dielectric tunability of BST films with 4 nm, 8 nm, 15 nm, and 40 nm thick TiO_2_ buffer layers are 46.9%, 60.8%, 39.3%, and 19.1%, respectively, while the tunability of the BST films without a buffer layer is 30.1%, as shown in Figure 7f. An ultra-thin (8 nm) TiO_2_ anatase buffer layer can significantly enhance the dielectric tunability of BST films. As discussed in Section 3.2, the lattice strain of BST films decreases as the thickness of the TiO_2_ anatase buffer layer increases. The TiO_2_ buffer layer plays an important role in the deposition of texture BST films for obtaining minimal stress and low lattice distortion, leading to enhanced polarization along the (111) preferential orientation, which is similar to those reports on the effects of TiO_2_ buffer layers on other films [34,35,36,37,38,39]. It can be concluded that ultra-thin TiO_2_ anatase buffer layers can adhere well to the Pt surface, minimizing both surface energy and lattice distortion required for the growth of BST nuclei along the (111) preferential orientation, resulting in significant improvements in dielectric tunability.

**Figure 8:**
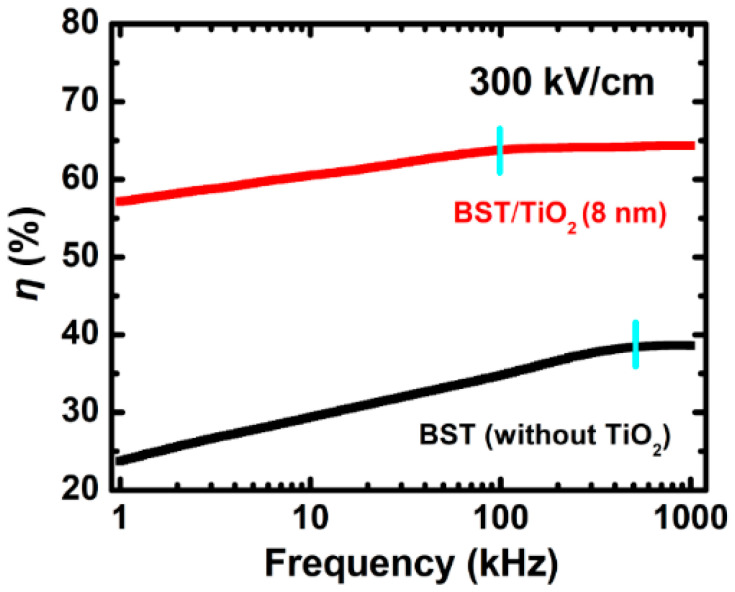
Dielectric tunability (*η*) as a function of the measuring frequency of BST films without and with an 8 nm TiO_2_ anatase buffer layer, respectively.

We further examined the dielectric tunability (*η*) variation tendency of BST films with the change in measurement frequency ranging from 1 kHz to 1 MHz at room temperature in detail. From Figure 8, for the BST films without TiO_2_, the dielectric tunability gradually increases with the increase in measurement frequency and then tends to saturate at 500 kHz (*η* = 38.6% at 1 MHz), which is similar to that in previous work [10]. The frequency dispersion of the dielectric tunability is large in this case. After incorporating an 8 nm TiO_2_ anatase buffer layer, the dielectric tunability of BST films significantly increases over the entire frequency measurement range (*η* = 64.3% at 1 MHz). It is noteworthy that the dielectric tunability saturation onset point position shifts to a low frequency (from 500 kHz to 100 kHz), and the frequency dispersion becomes relatively smaller, which makes great sense for achieving tunable microwave devices at high frequencies.

For comparison, here, we listed the reported dielectric properties of various BST thin films with different buffer layers in previous works [25,26,27,28,32,33], as shown in Table 3.

### 3.6. The I-V Characteristic Measurements

**Figure 9:**
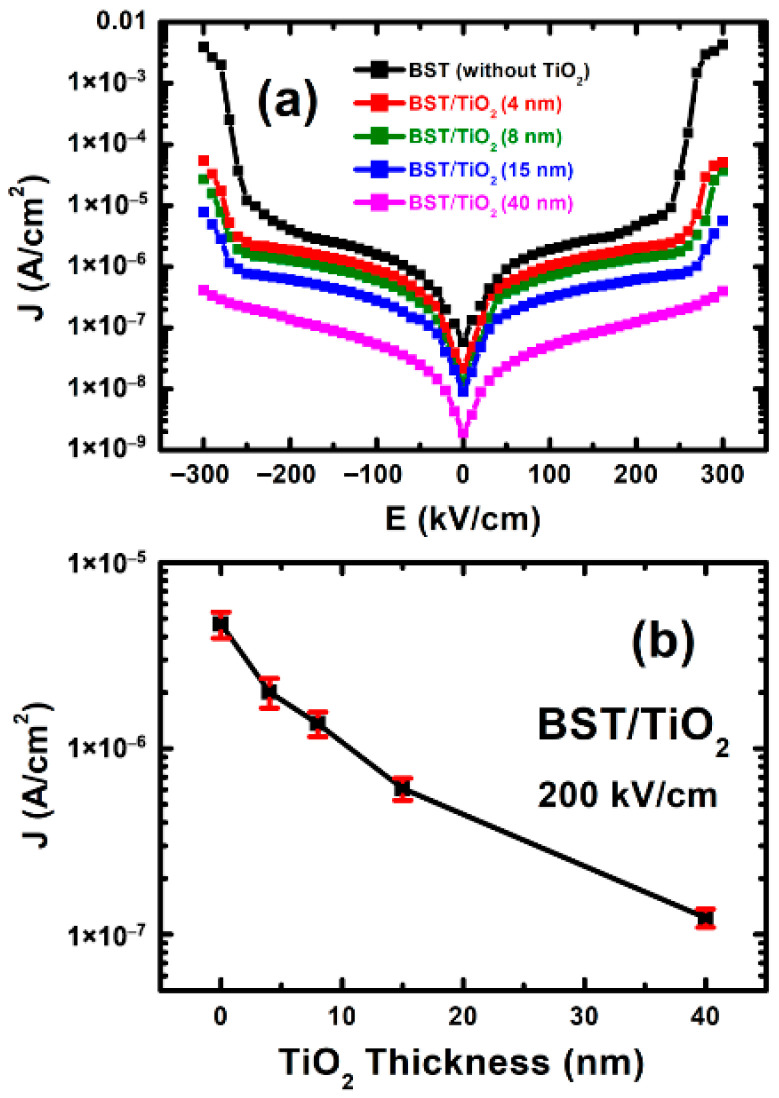
(**a**) Room-temperature I-V characteristics of BST films without and with 4 nm, 8 nm, 15 nm, and 40 nm TiO_2_ anatase buffer layers, respectively. (**b**) Leakage current densities (J) with a relative uncertainty of BST films vs. TiO_2_ thicknesses under 200 kV/cm.

Finally, we intensively investigated the *I-V* characteristics of the corresponding BST films within the applied electric field ranging from −300 kV/cm to 300 kV/cm. From Figure 9a, it can be observed that the leakage current density of BST films with a TiO_2_ buffer layer is lower than that of BST films without a buffer layer, demonstrating better insulation properties. All the leakage current densities with a relative uncertainty of corresponding BST films measured under 200 kV/cm are shown in Figure 9b and listed in Table 1. When an applied electric field of 200 kV/cm is used, the leakage currents of BST films without buffer layers and with 4 nm, 8 nm, 15 nm, and 40 nm TiO_2_ buffer layers are 4.67 × 10^−6^ A/cm^2^, 2.01 × 10^−6^ A/cm^2^, 1.36 × 10^−6^ A/cm^2^, 6.09 × 10^−7^ A/cm^2^, and 1.23 × 10^−7^ A/cm^2^, respectively.

It has been reported that the leakage current and dielectric loss of BST films largely depend on the surface roughness and decrease with a reduction in the surface roughness [46]. We found that due to the significant increase in the grain nucleation density and film density brought about by the addition of the TiO_2_ anatase buffer layer, the surface roughness of BST films is greatly improved. Consequently, both the leakage current and dielectric loss are correspondingly reduced. In addition, the breakdown voltage of BST films without buffer layer in Figure 9a is approximately 240 kV/cm. However, after adding an 8 nm thick TiO_2_ anatase buffer layer between Pt substrates and BST films, the breakdown voltage can increase to over 280 kV/cm. This indicates that an ultra-thin TiO_2_ buffer layer helps to enhance the voltage resistance of the BST films.

**Figure 10:**
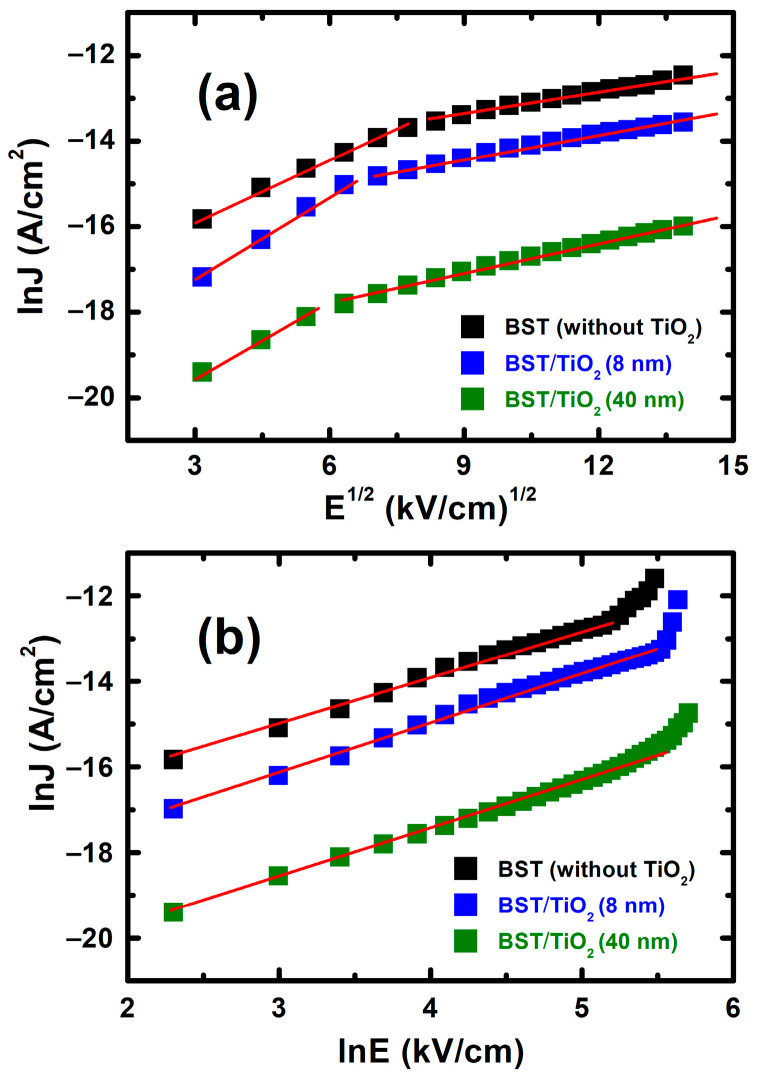
I-V characteristics of BST films without and with 8 nm and 40 nm TiO_2_ anatase buffer layers, (**a**) lnJ vs. E^1/2^ plots, and (**b**) lnJ vs. lnE plots, respectively.

To control excess leakage current in BST capacitors, we investigated the origin mechanisms of the leakage current in depth. Figure 10 shows lnJ vs. E^1/2^ plots and lnJ vs. lnE plots of I-V characteristics in BST films without and with 8 nm and 40 nm TiO_2_ anatase buffer layers, respectively. There are various mechanisms for the origin of leakage current in BST films, such as Schottky emission, Poole–Frenkel emission, space-charge limiting current (SCLC), Fowler–Northeim tunneling, etc. [13,30,31]. We first ruled out the possibility of the Poole–Frenkel emission mechanism, since all measurements were performed at room temperature. Generally, at the interface between electrodes (metal) and dielectric materials, charge carriers with low concentrations form Schottky barriers, and leakage currents are caused by Schottky emissions. In addition, if the number of carriers is very large, the Schottky barrier manifests as ohmic contact, and the leakage current is dominated by the SCLC mechanism. From Figure 10a, we found that the lnJ vs. E^1/2^ plot can be composed to two linear regions, indicating the existence of Schottky emission for both BST films without and with TiO_2_ anatase buffer layers. However, as shown in Figure 10b, the lnJ vs. lnE plots show that linear variation only occurs in the lower field region (E < 160 kV/cm for BST films without TiO_2_, and E < 220 kV/cm for BST films with a TiO_2_ anatase buffer layer), which can be considered as a SCLC mechanism. Thus, for BST films without TiO_2_, both Schottky emission and the SCLC mechanism contribute to leakage currents when E < 160 kV/cm, while for E > 160 kV/cm, Schottky emission plays a major role in leakage current. For BST films with a TiO_2_ anatase buffer layer, the turning point position shifts to a higher field region (from 160 kV/cm to 220 kV/cm), indicating that both Schottky emission and the SCLC mechanism contribute to leakage currents when E < 220 kV/cm, while for E > 220 kV/cm, Schottky emission plays a major role in leakage current for BST films with a TiO_2_ anatase buffer layer.

## 4. Conclusions

In summary, we systematically investigated the influence of TiO_2_ buffer layer thicknesses and preparation temperatures on the microstructure and electric properties of Ba_0.4_Sr_0.6_TiO_3_ films. We found that ultra-thin anatase crystalline TiO_2_ buffer layers within 15 nm thickness could significantly change BST films from irregular orientation to (111) preferential orientation. The dielectric constant was relatively increased while the dielectric loss and leakage current density were greatly reduced after the addition of the ultra-thin TiO_2_ anatase buffer layer. After incorporating an 8 nm TiO_2_ anatase buffer layer, the dielectric tunability of BST films significantly increased to 64.3% at 1 MHz, and the related frequency dispersion became relatively smaller over the entire frequency measurement range. The high tunable characteristics of BST films obtained by adding ultra-thin TiO_2_ anatase buffer layers and related research have important scientific significance and application value, which makes great sense for achieving tunable microwave devices at high frequencies.

## Figures and Tables

**Table 1:** Summary of the fabricating parameters, dielectric constant (ε_r_) and loss (tanδ) at 10 kHz, dielectric tunability (*η*) at 10 kHz under 300 kV/cm, and leakage current density (J) under 200 kV/cm.

Thin Film Type	TiO_2_ Thickness (nm)	Grain Size (nm)	Lattice Parameter (Å)	RMS Surface Roughness (nm)	ε_r_	tanδ	J (A/cm^2^)	*η* (%)
BST	0	42.25	3.9670	9.37	502.6	0.018	4.67 × 10^−6^	30.1
BST/TiO_2_	4	44.27	3.9681	7.76	700.3	0.013	2.01 × 10^−6^	46.9
BST/TiO_2_	8	46.69	3.9695	6.03	920.3	0.010	1.36 × 10^−6^	60.8
BST/TiO_2_	15	47.08	3.9744	4.59	622.7	0.008	6.09 × 10^−7^	39.3
BST/TiO_2_	40	48.46	3.9792	3.71	441.5	0.004	1.23 × 10^−7^	19.1

**Table 2:** Peak positions, *F.W.H.M.*, Lotgering orientation factors (F), and lattice strain along the (111) preferential orientation of BST films-buffered TiO_2_ with different thicknesses from XRD.

Film Type	Peak Position (deg.)			*F.W.H.M.* (deg.)			F			Lattice Strain
	(110)	(111)	(211)	(110)	(111)	(211)	(110)	(111)	(211)	
BST	31.821	39.235	56.690	0.201	0.199	0.230	—	—	—	0.0026
BST/TiO_2_ (4 nm)	31.809	39.219	56.687	0.204	0.191	0.232	−0.477	0.562	−0.019	0.0024
BST/TiO_2_ (8 nm)	31.798	39.203	56.677	0.207	0.181	0.233	−0.428	0.507	−0.018	0.0024
BST/TiO_2_ (15 nm)	31.744	39.183	56.614	0.212	0.179	0.238	−0.399	0.363	−0.013	0.0022
BST/TiO_2_ (40 nm)	31.712	39.150	56.613	0.216	0.174	0.242	−0.354	0.224	−0.009	0.0021

**Table 3:** Dielectric properties of various BST thin films with different buffer layers.

Materials/Buffer	Methods	Buffer Thickness (nm)	Frequency (Hz)	*E* (kV/cm)	*ε*_r_	*tan*δ	*η* (%)	Ref.
Ba_0.6_Sr_0.4_TiO_3_/MgO	PLD	10	1 M	300	275	0.009	30.0	[25]
Ba_0.7_Sr_0.3_TiO_3_/Bi	EBE	10	1 M	500	290	0.048	55.2	[26]
Ba_0.6_Sr_0.4_TiO_3_/LSCO	PLD	100	1 M	230	1170	0.01	75.0	[27]
Ba_0.3_Sr_0.7_TiO_3_/TiN	PLD	0.9	8 G	200	—	0.03	35.5	[28]
Ba_0.6_Sr_0.4_TiO_3_/TiO_2_	PLD	50	1 M	200	—	0.03	72.9	[32]
Ba_0.6_Sr_0.4_TiO_3_/Ta_2_O_5_	PLD	50	1 M	200	—	0.04	53.1	[32]
Ba_0.6_Sr_0.4_TiO_3_/TiO_2_	ALD	50	2 G	40	230	0.079	33.2	[33]
Ba_0.6_Sr_0.4_TiO_3_/TiO_2_	PLD	8	1 M	300	856.5	0.017	64.3	this work

## Data Availability

Data available on request to authors.
